# CNS *Neoscytalidium dimidiatum*: A Rare Cause of Fungal Brain Abscess

**DOI:** 10.1155/cris/4412140

**Published:** 2026-10-21

**Authors:** Abdulrahman M. Khojah, Ali Alkhaibary, Fahd AlSufiani, Ali H. Alassiri, Fadi Almahariq

**Affiliations:** ^1^ College of Medicine King Saud bin Abdulaziz University for Health Sciences Riyadh Saudi Arabia ksau-hs.edu.sa; ^2^ King Abdullah International Medical Research Center Riyadh Saudi Arabia kaimrc.med.sa; ^3^ Division of Neurosurgery Department of Surgery King Abdulaziz Medical City Ministry of National Guard – Health Affairs Riyadh Saudi Arabia mngha.med.sa; ^4^ Department of Pathology and Laboratory Medicine King Abdulaziz Medical City Ministry of National Guard – Health Affairs Riyadh Saudi Arabia mngha.med.sa

**Keywords:** brain abscess, central nervous system, fungal infection, immunocompromised, *Neoscytalidium dimidiatum*, phaeohyphomycosis

## Abstract

Neoscytalidium dimidiatum is a dematiaceous fungus that primarily affects plants but can rarely act as an opportunistic pathogen in humans, particularly in immunocompromised patients. It typically causes superficial skin and nail infections in tropical regions; however, central nervous system (CNS) infections are extremely rare. The neurotropism of this fungus is not well understood, but hematogenous spread from cutaneous or pulmonary foci has been hypothesized. A 60‐year‐old male with chronic lymphocytic leukemia (CLL) treated with acalabrutinib, a second‐generation Bruton’s tyrosine kinase (BTK) inhibitor, presented with a 3‐day history of personality changes, tension‐type headache, and progressive right‐sided weakness accompanied by dizziness. Physical examination revealed right hemiplegia and homonymous hemianopia. Brain MRI demonstrated a left parietal multiloculated ring‐enhancing intra‐axial lesion measuring 3.5 × 2 × 2.8 cm^3^, exerting mass effect on the ipsilateral lateral ventricle and causing rightward midline shift, with associated vasogenic edema and diffusion restriction. Given the immunocompromised state and atypical imaging findings, the patient underwent a left parietal stereotactic aspiration of the lesion. Histopathological examination using Grocott’s methenamine silver (GMS) staining revealed numerous septate, branching hyphae within the brain parenchyma and vessel walls, confirming an angioinvasive fungal infection. Serum beta‐D‐glucan was markedly elevated at 238 pg/mL, while galactomannan was negative. Fungal culture confirmed Neoscytalidium dimidiatum, identified based on colony morphology, microscopic characteristics, arthroconidia formation, and matrix‐assisted laser desorption/ionization time‐of‐flight (MALDI‐TOF) mass spectrometry. Dual antifungal therapy was initiated with intravenous liposomal amphotericin B and voriconazole, with subsequent dose escalation and route conversion. The patient was followed for ~12 months and survived with residual right‐sided motor deficits. The present case underscores the importance of early diagnosis, prompt surgical management, and appropriate antifungal therapy in improving outcomes for patients with CNS Neoscytalidium dimidiatum infection, particularly in the context of hematological malignancy and BTK‐inhibitor‐associated immunosuppressive therapy.

## 1. Introduction

*Neoscytalidium dimidiatum* (previously classified as *Scytalidium dimidiatum*, *Hendersonula toruloidea*, and *Nattrassia mangiferae*) is a dematiaceous, melanin‐pigmented fungus with significant taxonomic revisions reflecting its complex phylogeny [1]. Primarily a plant pathogen, it acts as an opportunistic human pathogen, causing superficial skin and nail infections in tropical regions. Central nervous system (CNS) infections are rare but devastating, with high mortality reported in published cases [2–8]. Neurotropism is poorly understood, but hematogenous dissemination from cutaneous or pulmonary foci is hypothesized and is often facilitated by a weakened immune system. Immunocompromised patients—particularly those with hematological malignancies, solid organ transplant recipients, and patients receiving targeted immunosuppressive therapies—are at elevated risk for invasive mold infections.

CNS phaeohyphomycosis poses a significant diagnostic challenge owing to its nonspecific clinical and radiological presentation, which overlaps with bacterial abscess, primary CNS lymphoma, and metastatic disease [9]. The present article describes a case of CNS *Neoscytalidium dimidiatum* infection by outlining the clinical presentation, radiological and pathological findings, management strategy, and outcomes, alongside a comparative overview of previously published cases. The comparative literature was identified through a structured search of PubMed, Scopus, and Embase using the terms “*Neoscytalidium dimidiatum*” AND (“central nervous system” OR “CNS”), with no date restriction; this search identified seven previously published cases of CNS *Neoscytalidium dimidiatum* infection, which are summarized alongside the present case in Table 1.

**Table 1:** Comparative summary of reported cases of CNS infections caused by *Neoscytalidium dimidiatum*.

Author	Age/sex	Underlying conditions	Clinical presentation	Diagnostic method	Treatment	Outcome
Alamri et al. [2]	55/F	Renal transplant, ATG	Fever, tonic‐clonic seizures, neurological deterioration	Brain biopsy: hyphae with angioinvasion; culture: *N. dimidiatum*, arthroconidia; ITS sequencing	Liposomal amphotericin B, voriconazole	Death
Geramishoar et al. [3]	17/M	SLE, prednisolone, cyclophosphamide	Seizure, fever, neck rigidity, drowsiness	Histopathology: hyphae; culture: Nattrassia mangiferae, arthroconidia	Amphotericin B	Death
Jo et al. [4]	62/M	Liver cirrhosis, HCC, diabetes	Dysarthria, general weakness	Aspirate culture: *N. dimidiatum*, arthroconidia; ITS sequencing	Amphotericin B, voriconazole	Survived
Dolatabadi et al. [5]	32/M	COVID‐19, tocilizumab, remdesivir	Fever, seizures, neurological symptoms	CSF culture: *N. dimidiatum*, arthroconidia; ITS sequencing; PAS: septate hyphae	Liposomal amphotericin B	Death
Mani et al. [6]	18/M	Immunocompetent	Headache, fever, vomiting, right‐sided weakness	Histopathology: hyphae; culture: *S. dimidiatum*, arthroconidia; DNA sequencing	Amphotericin B, fluconazole, posaconazole	Death
Alabdely et al. [7]	36/M	Renal transplant	Left hemiplegia	Histopathology and culture: *N. dimidiatum*, arthroconidia	Amphotericin B	Death
Alabdely et al. [7]	55/F	Renal transplant	Fever, convulsions	Histopathology, culture, molecular sequencing: *N. dimidiatum*, arthroconidia	Amphotericin B, voriconazole	Death
Tan et al. [8]	31/M	Renal transplant	Neurological deterioration	CSF culture: *S. dimidiatum*, arthroconidia; DNA sequencing	Itraconazole (empirical)	Death
Present case	60/M	CLL RAI III, acalabrutinib (BTK inhibitor), HTN, DLP, IHD, HBcAb‐positive	Personality change, headache, right hemiplegia, homonymous hemianopia, dizziness	GMS: angioinvasive septate hyphae; culture: *N. dimidiatum*, arthroconidia; MALDI‐TOF	Liposomal amphotericin B 315 mg/day (escalated to 10 mg/kg/day) + IV voriconazole 400 mg/day (switched to oral 200 mg q12h); left stereotactic aspiration	Survived (12 months); residual right hemiparesis (RUL 2/5, RLL 4/5)

Abbreviations: AmB, amphotericin B; ATG, antithymocyte globulin; BTK, Bruton’s tyrosine kinase; CLL, chronic lymphocytic leukemia; CSF, cerebrospinal fluid; DLP, dyslipidemia; GMS, Grocott’s methenamine silver; HBcAb, hepatitis B core antibody; HCC, hepatocellular carcinoma; HTN, hypertension; IHD, ischemic heart disease; ITS, internal transcribed spacer; MALDI‐TOF, matrix‐assisted laser desorption/ionization time‐of‐flight; NS, not specified; PAS, periodic acid‐Schiff; RLL, right lower limb; RUL, right upper limb; SLE, systemic lupus erythematosus.

## 2. Case Presentation

A 60‐year‐old male with chronic lymphocytic leukemia (CLL; Rai stage III) treated with acalabrutinib, a second‐generation Bruton’s tyrosine kinase (BTK) inhibitor, hypertension, dyslipidemia, ischemic heart disease (status post percutaneous coronary intervention), and hepatitis B core antibody positivity presented with personality changes, tension‐type headache, and progressive right‐sided weakness for 3 days. These symptoms were accompanied by dizziness. There was no history of seizures or documented fever.

Physical examination revealed right‐sided hemiplegia and homonymous hemianopia, with impaired gait. Brain MRI demonstrated a left parietal multiloculated ring‐enhancing intra‐axial lesion measuring ~3.5 × 2 × 2.8 cm^3^, with mass effect on the ipsilateral lateral ventricle and right‐sided midline shift of 0.7 cm, associated with diffuse vasogenic edema on FLAIR and a ring of diffusion restriction on DWI (Figure 1).

**Figure 1:**
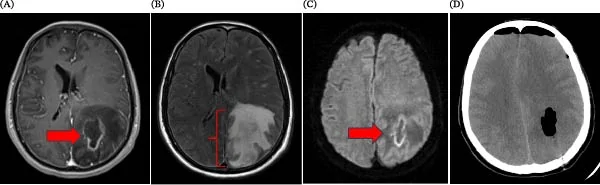
(A) Axial brain MRI with contrast showing a left parietal ring‐enhancing lesion measuring ~3.5 × 2 × 2.8 cm^3^ (arrow). The lesion causes mass effect on the ipsilateral lateral ventricle with a right‐sided midline shift of 0.7 cm. (B) FLAIR MRI demonstrating diffuse vasogenic edema surrounding the lesion (bracket). (C) DWI MRI showing a ring of diffusion restriction at the lesion margins (arrow). (D) Postoperative CT following left stereotactic aspiration of the abscess, demonstrating complete aspiration of the lesion and resolution of vasogenic edema.

At presentation, relevant laboratory parameters included hemoglobin 106 g/L, white blood cell count 22.30 × 10^3^/µL, platelets 233 × 10^3^/µL, creatinine 122 µmol/L, alanine aminotransferase (ALT) 11 U/L, aspartate aminotransferase (AST) 22 U/L, C‐reactive protein (CRP) 50.80 mg/L, and lactate 0.63 mmol/L. An extensive infectious workup was negative, including herpes simplex virus 1, varicella‐zoster virus, HHV‐6, CMV PCR (qualitative and quantitative), SARS‐CoV‐2, *Listeria monocytogenes*, *Streptococcus pneumoniae*, QuantiFERON‐TB Gold, and all carbapenemase resistance gene screens (IMP1, VIM, NDM, KPC, and OXA‐48). Toxoplasma IgG was 27.70 IU/mL, with IgM 3.00 IU/mL, interpreted as consistent with prior exposure rather than active disease. Chest X‐ray was unremarkable. No extracranial source of fungal infection was identified. On the day of aspiration, serum beta‐D‐glucan was markedly elevated at 238.0 pg/mL, while serum galactomannan was negative, strongly supporting an invasive non‐*Aspergillus* mold infection.

Given the immunocompromised state, atypical imaging findings, and concern for infectious versus neoplastic etiology, urgent tissue diagnosis was pursued. The patient underwent a left parietal stereotactic aspiration of the lesion. A burr hole was created in the left parietal cranium, and a needle biopsy was advanced under stereotactic guidance into the target lesion. A reddish‐brown, soft‐consistency material was aspirated and collected. Specimens were submitted simultaneously for intraoperative frozen section, formal hematoxylin and eosin (H&E) and Grocott’s methenamine silver (GMS) histopathological examination, fungal culture, tuberculosis polymerase chain reaction (TB PCR), and microbiological cultures.

Intraoperative frozen section demonstrated reactive inflammation with gliosis and lymphocytic infiltration. Formal H&E sections of the brain tissue showed a mixed inflammatory infiltrate including neutrophils, plasma cells, and small mature‐looking lymphocytes, with reactive gliosis and scattered karyorrhectic debris within the brain parenchyma (Figure 2A). GMS staining (Figure 2B) further highlighted numerous septate, branching fungal hyphae within the brain parenchyma and vessel walls, confirming angioinvasive fungal infection. Fungal culture from the aspiration specimen yielded *Neoscytalidium dimidiatum*, identified on the basis of colony morphology—multiple greenish‐pigmented colonies with a whitish wooly surface appearance (Figure 3A)—microscopic characteristics including septate hyphae and arthroconidia formation (Figure 3B), and matrix‐assisted laser desorption/ionization time‐of‐flight (MALDI‐TOF) mass spectrometry for species identification.

**Figure 2:**
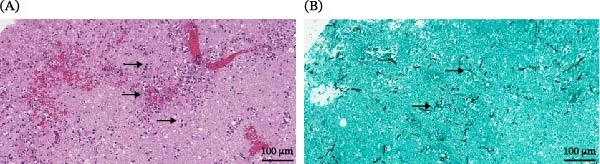
(A) Hematoxylin and eosin stain showing brain parenchyma with inflammatory infiltrate including neutrophils, plasma cells, and small mature‐looking lymphocytes. There is reactive gliosis with areas of necrosis and scattered karyorrhectic debris within the brain parenchyma (arrows). (B) Special staining (GMS) further highlights numerous septate, branching fungal hyphae within the brain parenchyma and vessels (arrow). The tissue culture confirmed *Neoscytalidium dimidiatum*.

**Figure 3:**
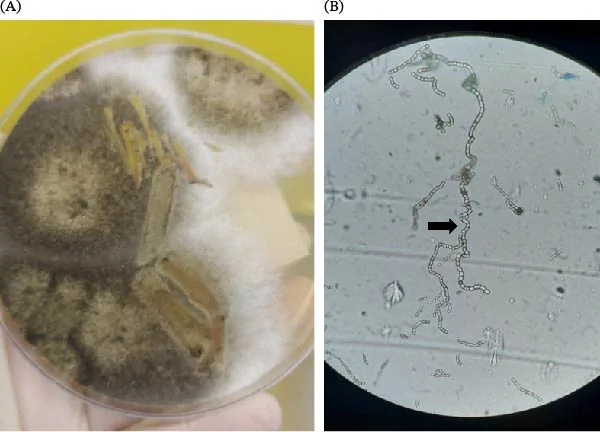
(A) Fungal culture of *Neoscytalidium dimidiatum* following incubation, showing multiple greenish‐pigmented colonies with a whitish wooly appearance. (B) Microscopic examination of the cultured fungus showing multiple septate hyphae and arthroconidia formation.

Following surgical source control, corticosteroid therapy was administered for management of perilesional vasogenic edema, comprising dexamethasone 4 mg intravenously every 6 h (16 mg/day equivalent), initiated postbiopsy and maintained for several days before a gradual taper. Upon confirmation of fungal infection, dual antifungal therapy was initiated: intravenous liposomal amphotericin B, initiated at 315 mg/day (~4–5 mg/kg/day) and subsequently escalated to 10 mg/kg/day following definitive species identification and surgical source control, and intravenous voriconazole, initiated at 400 mg/day and later transitioned to an oral formulation at 200 mg every 12 h for extended outpatient therapy. Dual intravenous therapy was continued for ~6 weeks in the hospital, with oral voriconazole maintained thereafter for a planned extended course of at least 12 months, guided by serial clinical and radiological response. Prophylactic cotrimoxazole (160 mg orally) was maintained throughout for *Pneumocystis jirovecii* prophylaxis.

Over the course of antifungal therapy, serial laboratory monitoring documented expected treatment‐related trends, including a hemoglobin nadir of 63 g/L (attributed to a combination of CLL‐related marrow infiltration and phlebotomy losses, requiring transfusion support), a platelet nadir of 18 × 10^3^/µL (attributed to CLL‐related thrombocytopenia and myelosuppressive effects of concurrent therapy), a creatinine peak of ~270 µmol/L (attributed to amphotericin B‐associated nephrotoxicity, prompting dose adjustment and intensified hydration), an ALT peak of 77 U/L (attributed to combined antifungal hepatotoxicity), and a potassium nadir of 2.5 mmol/L (attributed to amphotericin B‐associated renal tubular wasting, managed with electrolyte repletion). These derangements improved with supportive management and dose adjustment without requiring the discontinuation of antifungal therapy.

Serial follow‐up MRIs documented progressive radiological improvement. The patient remains alive at ~12 months of follow‐up. At the latest follow‐up, the patient had a baseline Glasgow Coma Scale (GCS) of 14/15 with residual right‐sided motor deficits (right upper limb power 2/5 and right lower limb power 4/5). The patient continues regular clinical and radiological follow‐up.

## 3. Discussion

*Neoscytalidium dimidiatum* is a melanized (dematiaceous) mold responsible for a rare but severe form of CNS infection in immunocompromised hosts. High mortality has been reported in published cases of CNS *Neoscytalidium dimidiatum* infection [2–8]; only one previously reported patient survived. The present case represents the first reported case of invasive CNS *Neoscytalidium dimidiatum* infection in a patient with CLL and the longest documented survival in the literature at 12 months.

The differential diagnosis of ring‐enhancing brain lesions in immunocompromised patients is broad and includes bacterial brain abscess, toxoplasmosis, primary CNS lymphoma, metastatic malignancy, and other invasive fungal infections [9]. In this patient, the initial leading differential included CNS CLL involvement given Rai stage III disease. Toxoplasmosis was considered given the mildly elevated Toxoplasma IgG (27.70 IU/mL), but the borderline IgM (3.00 IU/mL) and clinical context favored prior exposure rather than active disease. Bacterial etiologies were excluded by negative blood and tissue cultures and a comprehensive multidrug‐resistant organism (MDRO) screen. Negative serum galactomannan effectively excluded *Aspergillus* species, while the markedly elevated beta‐D‐glucan (238 pg/mL) supported an invasive mold infection. Histopathological demonstration of angioinvasive, septate, branching hyphae on GMS staining—a morphology distinct from the nonseptate or pauciseptate hyphae typically observed with Mucorales—was most consistent with a dematiaceous mold, subsequently confirmed as *Neoscytalidium dimidiatum* on culture. The finding of fungal hyphae within vessel walls confirmed angioinvasion, a cardinal histopathological feature of phaeohyphomycosis caused by melanized molds that likely accounts for the hemorrhagic foci and mass effect observed radiologically in this and other published cases [1, 9].

Patients with CNS infections caused by *Neoscytalidium dimidiatum* typically present with fever, seizures, and focal neurological deficits such as hemiplegia or weakness; headache, vomiting, neck rigidity, and altered mental status have also been reported [2–8]. The onset is often acute, with rapid deterioration. For instance, one patient developed tonic‐clonic seizures and neurological decline within a month posttransplant [2], and another presented with seizures and drowsiness in the setting of systemic lupus erythematosus [3]. In the present case, the absence of fever combined with the insidious onset of personality change, headache, and hemiplegia in an immunocompromised host underscores the clinical variability of this infection and the need for a high index of suspicion. CNS infections caused by *Neoscytalidium dimidiatum* are radiologically characterized by single or multiple abscesses, ring‐enhancing lesions, hydrocephalus, and subacute infarctions, identifiable by CT or MRI [2–8].

Published cases of CNS *Neoscytalidium dimidiatum* uniformly involve severely immunocompromised patients, including solid organ transplant recipients on immunosuppressive agents [2, 7, 8]. A single case involving an immunocompetent host has been reported [6]. The present case expands the spectrum of predisposing conditions to include CLL and, notably, targeted BTK‐inhibitor therapy.

Acalabrutinib, the BTK inhibitor administered to this patient for CLL, is increasingly recognized as an independent risk factor for invasive fungal infections, including invasive aspergillosis and other non‐*Aspergillus* mold infections, several of which have involved the CNS [10, 11]. BTK is required for Dectin‐1‐ and Toll‐like receptor‐mediated NF‐κB and NFAT signaling in monocytes and macrophages, and BTK inhibition impairs the innate antifungal response of these cells, independent of the underlying lymphoid malignancy or neutrophil count [12]. This mechanism is consistent with the absence of frank neutropenia in the present case (white blood cell count 22.30 × 10^3^/µL with lymphocytic predominance) despite the development of an aggressive, angioinvasive CNS mold infection and suggests that BTK‐inhibitor‐associated functional immune deficiency, rather than CLL‐related cytopenia alone, was a principal driver of susceptibility in this patient. Clinicians managing patients on BTK inhibitors should therefore maintain heightened vigilance for invasive mold infections, including rare non‐*Aspergillus* organisms such as *Neoscytalidium dimidiatum*, even in the absence of neutropenia or other classical risk factors.

Treatment of CNS *Neoscytalidium dimidiatum* requires aggressive multimodal therapy encompassing surgical source control and prolonged dual‐antifungal therapy. In this case, the voriconazole minimum inhibitory concentration (MIC) was 0.5 µg/mL (0.125 µg/mL on repeat), and the amphotericin B MIC was 0.5 µg/mL. No standardized susceptibility breakpoints exist for Neoscytalidium species, and in vitro MIC values have not reliably predicted clinical response in previously published cases; these values are therefore reported descriptively rather than as a guide to expected treatment efficacy. The rationale for dual antifungal therapy was the severity of CNS involvement, confirmed angioinvasive pathology, multiloculated abscess morphology, and susceptibility profile. Voriconazole was selected for its excellent CNS penetration, while liposomal amphotericin B provided complementary antifungal activity against melanized molds.

Species‐level identification in the present case relied on colony morphology, microscopic characteristics (septate hyphae and arthroconidia formation), and MALDI‐TOF mass spectrometry. Internal transcribed spacer (ITS) rDNA sequencing, which was used for molecular confirmation in several previously published cases [2, 4, 5, 8], was not performed at our institution at the time of culture processing. This represents a limitation of the present report, as morphological and MALDI‐TOF‐based identification, while consistent with the phenotypic characteristics of *Neoscytalidium dimidiatum* described in the literature, lacks the definitive discriminatory power of molecular sequencing for closely related dematiaceous species.

Among all seven previously published cases of CNS *Neoscytalidium dimidiatum* [2–8], only one patient survived [4]. The present patient survived at 12 months, though with significant residual neurological morbidity, including right hemiparesis (upper limb 2/5 and lower limb 4/5). This outcome highlights that survival is achievable with early diagnosis, aggressive multimodal surgical and antifungal management, and sustained clinical monitoring; however, it may be associated with substantial functional disability. Clinicians should maintain a high index of suspicion for rare mold infections in immunocompromised patients presenting with ring‐enhancing CNS lesions—particularly those receiving BTK‐inhibitor therapy—initiate early tissue biopsy for definitive diagnosis, and consider fungal etiologies in the differential from the outset.

**Nomenclature**
CNS: Central nervous system
MRI: Magnetic resonance imaging
FLAIR: Fluid‐attenuated inversion recovery
DWI: Diffusion‐weighted imaging
CT: Computed tomography
H&E: Hematoxylin and eosin
GMS: Grocott’s methenamine silver
CSF: Cerebrospinal fluid
ATG: Antithymocyte globulin
SLE: Systemic lupus erythematosus
HCC: Hepatocellular carcinoma
NS: Not specified
BTK: Bruton’s tyrosine kinase
CLL: Chronic lymphocytic leukemia
MIC: Minimum inhibitory concentration
CRP: C‐reactive protein
AKI: Acute kidney injury
MALDI‐TOF: Matrix‐assisted laser desorption/ionization time‐of‐flight
GCS: Glasgow Coma Scale
TB PCR: Tuberculosis polymerase chain reaction
MDRO: Multidrug‐resistant organism
ITS: Internal transcribed spacer.

## Funding

No funding was received for this study.

## Ethics Statement

Written informed consent for the publication of this case report and accompanying images was obtained from the patient’s legal representative. This report complies with the institutional ethics and research committee requirements of King Abdullah International Medical Research Center (KAIMRC), King Abdulaziz Medical City, Riyadh, Saudi Arabia.

## Conflicts of Interest

The authors declare no conflicts of interest.

## Data Availability

The data that support the findings of this study are available from the corresponding author upon reasonable request.

## References

[1] Revankar S. G. and Sutton D. A. , Melanized Fungi in Human Disease, Clinical Microbiology Reviews. (2010) 23, no. 4, 884–928, 10.1128/CMR.00019-10. PMC2952981 20930077

[2] Alamri M. , Alghamdi H. , and Althawadi S. , et al.Invasive Fungal Infection of the Brain Caused by *Neoscytalidium dimidiatum* in a Post-Renal Transplant Patient: A Case Report, Medical Mycology Case Reports. (2021) 34, 27–31, 10.1016/j.mmcr.2021.09.001. PMC8463796 34603948

[3] Geramishoar M. , Zomorodian K. , and Zaini F. , et al.First Case of Cerebral Phaeohyphomycosis Caused by *Nattrassia mangiferae* in Iran, Japanese Journal of Infectious Diseases. (2004) 57, no. 6, 285–286, 10.7883/yoken.JJID.2004.285. 15623958

[4] Jo S. Y. , Lee S. , Kim K. H. , and Yi J. , A Case of Brain Abscess Caused by the Dematiaceous Mold *Neoscytalidium dimidiatum* in a Korean Man, Annals of Laboratory Medicine. (2021) 41, no. 2, 247–249, 10.3343/alm.2021.41.2.247. PMC7591279 33063689

[5] Dolatabadi S. , Nasirharandi S. , and Pourahmad M. , et al.Phaeohyphomycosis Caused by *Neoscytalidium dimidiatum* in a COVID-19 Patient, Revista Iberoamericana De Micologia. (2022) 39, no. 3-4, 72–76, 10.1016/j.riam.2022.07.002. 36456391

[6] Mani R. S. , Chickabasaviah Y. T. , and Nagarathna S. , et al.Cerebral Phaeohyphomycosis Caused by *Scytalidium dimidiatum*: A Case Report From India, Medical Mycology. (2008) 46, no. 7, 705–711, 10.1080/13693780802172017. 18608894

[7] Alabdely M. H. , Alolayan A. S. , Almaghrabi R. S. , and Al-Abdely H. M. , Cerebral Phaeohyphomycosis at a Tertiary Healthcare Center in Saudi Arabia, Neurosciences. (2023) 28, no. 2, 136–142, 10.17712/nsj.2023.2.20220118. PMC10155477 37045456

[8] Tan D. H. S. , Sigler L. , Gibas C. F. C. , and Fong I. W. , Disseminated Fungal Infection in a Renal Transplant Recipient Involving *Macrophomina phaseolina* and Scytalidium Dimidiatum: Case Report and Review of Taxonomic Changes Among Medically Important Members of the Botryosphaeriaceae, Medical Mycology. (2008) 46, no. 3, 285–292, 10.1080/13693780701759658. 18404556

[9] Revankar S. G. , Sutton D. A. , and Rinaldi M. G. , Primary Central Nervous System Phaeohyphomycosis: A Review of 101 Cases, Clinical Infectious Diseases. (2004) 38, no. 2, 206–216, 10.1086/380635. 14699452

[10] Chamilos G. , Lionakis M. S. , and Kontoyiannis D. P. , Call for Action: Invasive Fungal Infections Associated With Ibrutinib and Other Small Molecule Kinase Inhibitors Targeting Immune Signaling Pathways, Clinical Infectious Diseases. (2018) 66, no. 1, 140–148, 10.1093/cid/cix687. PMC5850040 29029010

[11] Ghez D. , Calleja A. , and Protin C. , et al.Early-Onset Invasive Aspergillosis and Other Fungal Infections in Patients Treated With Ibrutinib, Blood. (2018) 131, no. 17, 1955–1959, 10.1182/blood-2017-11-818286. 29437588

[12] Fiorcari S. , Maffei R. , and Vallerini D. , et al.BTK Inhibition Impairs the Innate Response Against Fungal Infection in Patients With Chronic Lymphocytic Leukemia, Frontiers in Immunology. (2020) 11, 10.3389/fimmu.2020.02158, 2158. PMC7485008 32983178

